# Bottom-Up Realization of a Type-II Organic/Transition-Metal Dichalcogenides Heterointerface: Pentacene on Monolayer WS_2_

**DOI:** 10.1021/acsnano.6c07654

**Published:** 2026-09-04

**Authors:** Michele Capra, Christian S. Kern, Mira S. Arndt, Karl J. Schiller, Max Niederreiter, Francesco Presel, Iolanda Di Bernardo, Marco Gruenewald, Torsten Fritz, Stefan Tappertzhofen, Martin Sterrer, Peter Puschnig, Mirko Cinchetti, Giovanni Zamborlini

**Affiliations:** † Department of Physics, 14311 TU Dortmund University, 44227 Dortmund, Germany; ‡ Institute of Physics, NAWI Graz, 27267 University of Graz, 8010 Graz, Austria; § Institute of Solid State Physics, 9378 Friedrich Schiller University Jena, 07743 Jena, Germany; ∥ Department of Electrical Engineering and Information Technology, TU Dortmund University, 44227 Dortmund, Germany; ⊥ Universidad Universidad Autónoma de Madrid and IFIMAC, 28049 Madrid, Spain

**Keywords:** tungsten disulfide, organic−inorganic heterostructures, type-II band alignment, epitaxial growth, scanning tunneling microscopy, angle-resolved photoemission spectroscopy, density functional theory

## Abstract

Stacked van der Waals heterostructures based on transition-metal dichalcogenides (TMDs) exhibit a rich variety of exotic interfacial phenomena. Substituting one component with an organic semiconductor (OSC) enables the design of hybrid heterostructures with tunable functionalities for optoelectronic, photovoltaic, and spintronic applications. In this work, exploiting scanning tunneling spectroscopy (STS), photoemission orbital tomography (POT), and *G*_0_ *W*_0_ electronic structure calculations, we experimentally and theoretically demonstrate the self-assembly of an ordered single layer of pentacene (5A) above monolayer WS_2_, exhibiting a type-II (staggered) band alignment in the hybrid 5A/WS_2_ interface. Central to this result is the synthesis of extended, atomically flat WS_2_ — an essential prerequisite for a highly ordered and electronically homogeneous OSC/TMD interface — which can only be reliably achieved via bottom-up growth, most notably molecular beam epitaxy (MBE). We realize this by leveraging Au(111) as an atomically clean and conductive sample for epitaxial growth — a necessary requirement for reliable and comparable STS/POT characterizations. The high quality of the synthesized heterostructure, together with its type-II band alignment, establishes pentacene/WS_2_ as a model system for orbital-resolved studies of charge transfer, energy-level renormalization, and nonequilibrium interfacial processes in hybrid organic/inorganic 2D heterostructures.

## Graphical abstract

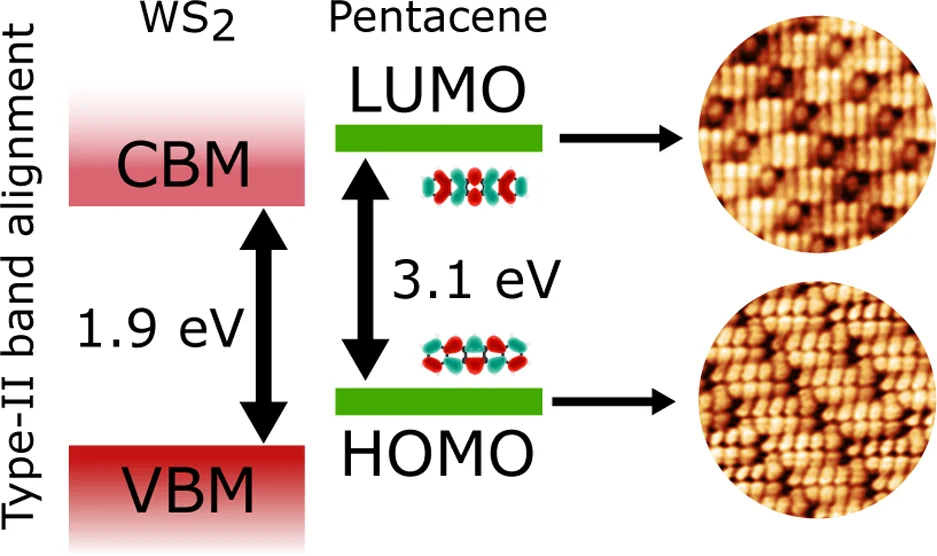

## Introduction

Transition-metal dichalcogenides (TMDs), a class of 2D materials where a single layer of transition-metal atoms is sandwiched between two layers of chalcogens (S, Se, or Te),^1,2^ have attracted significant scientific interest owing to their unique electronic and optical properties.

Depending on their composition, bulk TMDs can exhibit electronic properties ranging from metallic to semiconducting^1,2^ to Weyl semimetals^3^ (*e.g.*, WTe_2_) and display a variety of nontrivial electronic properties, including superconductivity^1,4,5^ (*e.g.*, NbSe_2_), charge density wave ordering^6^ (*e.g.*, TaS_2_), and spin valley splitting^1,7^ (*e.g.*, WS_2_). In semiconducting group IV TMDs, it is possible to control both the size and type of the band gap via their thickness.^8,9^ The band gap is inversely proportional to the TMD’s thickness, and, at the monolayer limit, an indirect-to-direct band gap transition also occurs.^1,10,11^ The plethora of these phenomena make TMDs suitable for the development of next-generation electronics, photonics, and optoelectronics applications,^12–15^ like photodetectors,^16,17^ solar cells,^18^ and transistors.^19^ Moreover, stacking together different 2D materials to form van der Waals heterostructures has been shown to induce new properties that do not arise from the individual constituents of the junctions, but rather from their interface.^20,21^ As an example, such interfaces can host interlayer excitons,^20^ where the electron and the hole are located in two separate materials (at the interface), or moiré states, resulting from twisting two different 2D materials.^21^

Being based on atomically thin materials, where each layer simultaneously acts as both bulk and interface, the surface quality plays a crucial role in the performance of 2D heterojunctions. Up to now, the most common approaches for the synthesis of heterojunctions have relied on top-down methods,^22^ like exfoliation of bulk crystals with tape or liquid sonication, which typically results in only micrometer-sized and poor-quality interface areas. Bottom-up approaches, though more demanding from a technical point of view, enable the synthesis of highly crystalline, almost defect-free, and millimeter-scale interfaces.^23,24^ Beyond structural advantages, these methods also open up unique opportunities for nanoscale functionalization, making them particularly promising for tailored molecular integration.^25–27^ In particular, hybrid interfaces between TMDs and organic semiconducting (OSC) molecules are gaining interest due to the ability of molecules to efficiently tune optical, electronic, and magnetic properties of 2D materials^28^ combined with a seamless implementation, low cost, and an almost infinite choice of organic molecules.

In this context, interfacing OSCs with TMDs holds the promise of coupling the high responsivity to external stimuli offered by OSCs with the superior charge transport of monolayer TMDs,^29,30^ as also supported by recent breakthroughs in the exciton-mediated optical control of proximity effects at organic/metal interfaces.^31,32^ Extending this concept to semiconducting heterojunctions unlocks new opportunities for photovoltaic, optoelectronic, and sensing applications. However, the performance of such hybrid systems critically depends on the relative alignment of molecular and TMD energy levels,^28,30^ which defines the direction and efficiency of charge transfer across the interface. Depending on the band alignment, three main heterojunction types can be identified: type I (straddling), type II (staggered), and type III (broken gap). Among those, type II architectures are particularly attractive as their staggered band alignment promotes spatial charge separation between the layers. Consequently, such interfaces may support the formation of long-lived interlayer excitons,^29^ enabling efficient energy transfer from the strongly light absorbing organic layer to the high-mobility TMD substrate.^29,31,32^

In this study, we synthesize a hybrid interface comprising single-layer WS_2_ and a single layer of pentacene (5A) that self-assembles atop the TMD, forming a long-range ordered film. WS_2_ was chosen over other commonly studied semiconducting TMDs, such as MoS_2_ and WSe_2_, because it exhibits the largest band gap among TMDs^33^ and it is exceptionally air-stabile compared to diselenides.^34^ The electronic and geometric properties of the interface have been revealed with a multitechnique approach that combines scanning tunneling microscopy (STM) and spectroscopy (STS), angle-resolved photoemission spectroscopy (ARPES), and photoemission orbital tomography (POT), supported by ab-initio electronic structure calculations at the density functional theory (DFT) and the *G*_0_ *W*_0_ level, respectively. Inspired by earlier studies on MoS_2_, NbS_2_, and VS_2_,^35–37^ we demonstrate here the first successful growth of extended, atomically flat WS_2_ single crystals using dimethyl disulfide (DMDS) as a sulfur source, an environmentally benign and nontoxic alternative to the commonly used H_2_S.^38,39^ Upon adsorption of the 5A layer, we observe the formation of a commensurate molecular superstructure characterized by type-II band alignment, in which the highest-occupied molecular orbital (HOMO) of 5A lies within the WS_2_ band gap, while the lowest-unoccupied molecular orbital (LUMO) overlaps with the WS_2_ conduction band. Our bottom-up approach not only ensures high crystalline quality but also provides clean, chemically accessible surfaces, crucial for the controlled assembly of molecular layers.

## Results and Discussion

First, we assess the chemical and crystalline quality of the WS_2_ grown atop Au(111) from the DMDS precursor (more details can be found in the Methods section). The chemical composition of the as-deposited WS_2_ monolayer has been checked by means of X-ray photoemission spectroscopy (XPS). Figure 1a,b shows the acquired core level spectra for W 4f and S 2p. The energy positions of W 4f_7/2_ and S 2p_3/2_ core levels (32.7 and 162.4 eV, respectively) are in excellent agreement with the reported values^40^ for a monolayer of WS_2_ grown on Au(111). Moreover, the lack of any detectable features at binding energies below 32.7 eV in Figure 1a proves the full sulfurization of tungsten atoms, excluding any significant substoichiometric species or W clusters.

**Figure 1:**
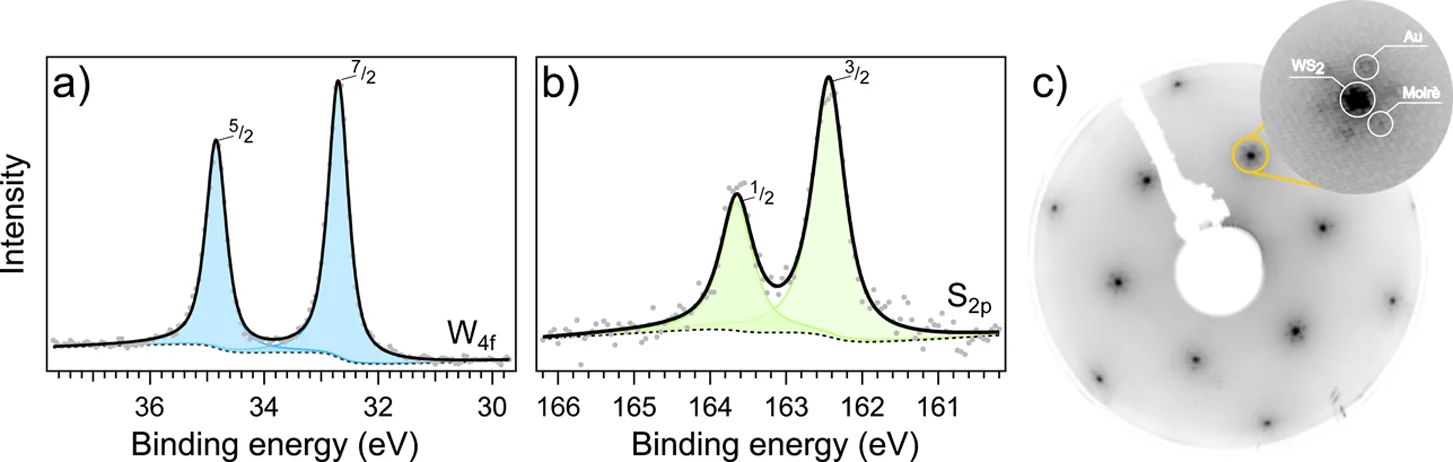
XPS spectra of monolayer WS_2_ measured at room temperature. (a) Tungsten 4f orbitals and (b) sulfur 2p orbitals. (c) Low-energy electron diffraction (LEED) pattern of the WS_2_/Au­(111) interface, showing the moiré pattern — inset — induced by slight mismatch in lattice constants for Au(111) and WS_2_.

The surface quality and crystallinity have been investigated by means of low-energy electron diffraction (LEED) and STM. The LEED pattern of the as-grown WS_2_ monolayer, shown in Figure 1c, displays a clear moiré pattern, surrounding the main diffraction spot of WS_2_. The inset in Figure 1c shows a close-up on the moiré pattern in which one of the outer points is superimposed to the gold LEED pattern.^40^ This moiré pattern is typical of many TMDs grown on Au(111),^36,37,40^ and it originates from the mismatch between the nominal lattice parameter^41^ of Au(111), 2.89 Å, and the one of the TMD. Its presence also serves as an indicator of the high crystalline quality of the TMD and as a proof of epitaxial growth.

Figure 2a,b show, respectively, a large-scale and an atomically resolved STM image of the WS_2_ monolayer, providing an overview of its surface morphology. Two distinct features can be clearly identified in Figure 2a: first, the moiré pattern also observed in the LEED measurements, and, second, a surface morphology characterized by randomly distributed depressions of varying sizes. These depressions, characterized by a height of a single Au(111) atomic step, are indicative of local structural inhomogeneities or possible growth-related defects.

**Figure 2:**
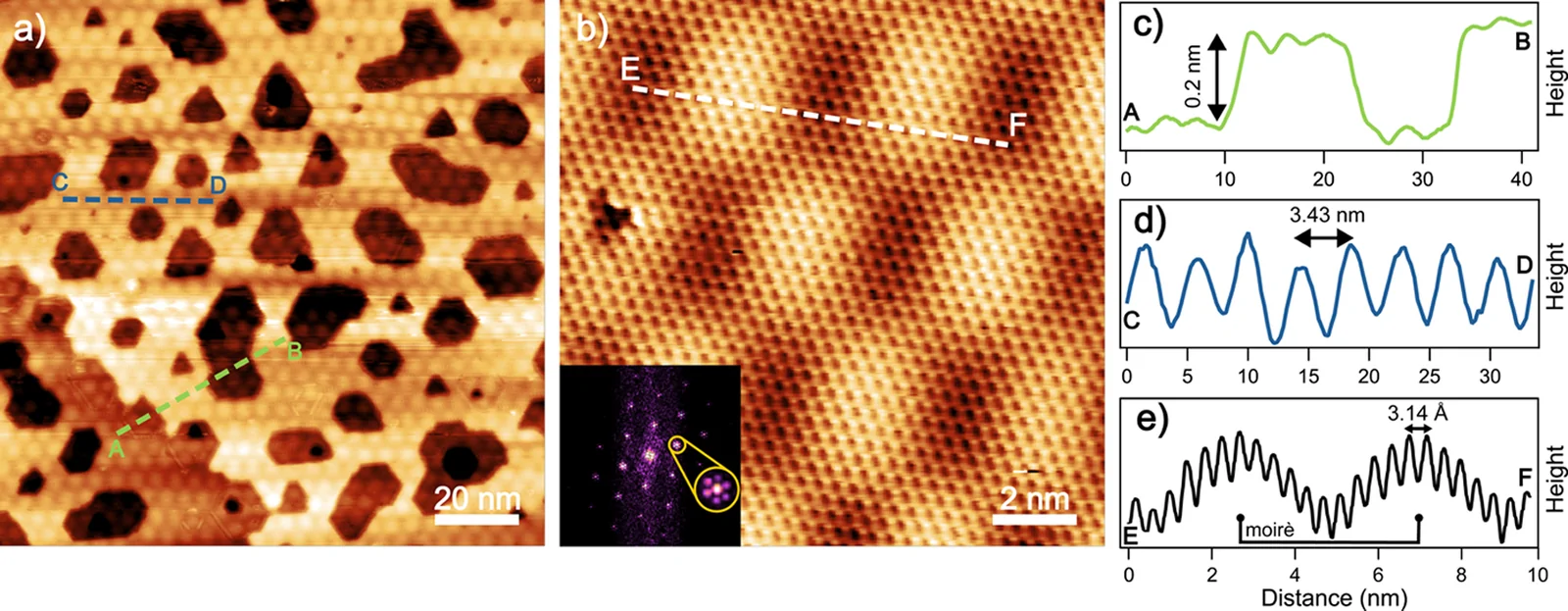
(a) Large-scale STM image of the WS_2_/Au­(111) interface; *V* = 0.084 V, *I* = 20 pA, (130 × 130) nm^2^, *T* = 77 K; (b) atomic resolution of WS_2_ monolayer; *V* = −1.4 V, *I* = 20 pA, (13 × 13) nm^2^, *T* = 4 K; the inset shows the fast Fourier transform of image (b) where moiré satellite spots are observed, as in the LEED pattern; (c) height profile across Au(111) terrace step and hole, consistent with the height of single layer of Au(111); (d) line profile showing the moiré periodicity; (e) line profile showing the lattice constant of WS_2_ superimposed to the moiré periodicity induced by the underneath Au(111).

From an STM line scan across a step edge (Figure 2c), we determine a step height of 0.2 nm, which is consistent with Au(111) atomic steps and thereby confirms the continuity of the WS_2_ film across the gold surface, see also Figure S1. The depression pattern which characterizes the entire surface has been already observed on gold surfaces exposed to high doses of sulfur:^42^ due to its high reactivity, sulfur corrodes the gold surface, yielding one-atom deep depressions (see line profile in Figure 2c), where the depth of the depression is seen to match the height of a substrate step edge. Nevertheless, we observe a continuous moiré pattern across boundaries of those depressions, meaning that WS_2_ forms a seamless film covering/carpeting the depressions as well as step edges, as also seen in Figure S1.

The measured periodicity associated with the moiré pattern is 3.43 nm, Figure 2d, while in Figure 2e, we report the line scan from the atomically-resolved image of the WS_2_ monolayer, Figure 2b, where it is possible to observe the moiré pattern superimposed on the atomic unit cell of WS_2_, measured as 0.314 nm and consistent with the lattice constant reported in literature.^43^ These parameters are also consistent with the moiré empirical formula $d_{moiré}=\frac{a_{Au}a_{{WS}_{2}}}{\left|a_{Au}-a_{{WS}_{2}}\right|}=3.48\;nm$ .

The measured values for the WS_2_/Au­(111) lattice constant and moiré periodicity of, respectively, 0.316 and 3.2 nm, reported in a previous work^43^ are in fair agreement with our values of 0.314 and 3.43 nm. Note that the slight differences also result in different commensurate moiré supercells. Our results suggest (11 × 11) for WS_2_ and (12 × 12) for Au(111), while the previously reported moiré supercell for WS_2_ and Au(111) were (10 × 10) and (11 × 11), respectively.

We now discuss the electronic properties of the WS_2_/Au­(111) interface, investigated by means of both ARPES and STS. In Figure 3a, we show the electronic band dispersion for the WS_2_ monolayer along the high symmetry directions indicated in Figure 3b. Clear spin-split bands at the high symmetry point ***K̅***, characteristic of both W and Mo based TMDs, confirm the high quality of the grown WS_2_ monolayer, with a measured band splitting of 417 meV, consistent with previously reported values.^44,45^ Since the growth of WS_2_ on metals does not follow a self-terminating protocol, the second layer is expected to nucleate^46^ already from coverages of 0.7 ML.

**Figure 3:**
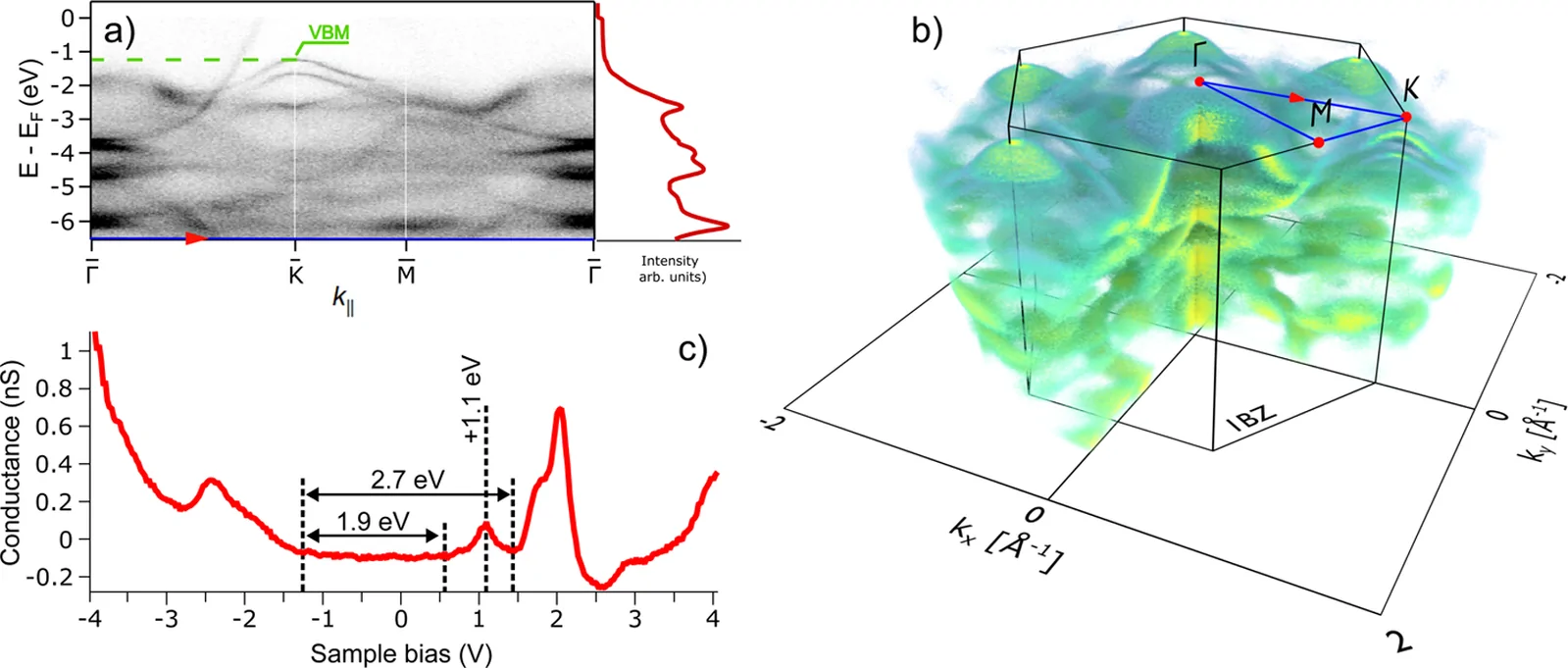
(a) ARPES spectra of monolayer WS_2_/Au­(111) along high symmetry directions in the Brillouin zone (shown in (b)). The characteristic spin split bands of WS_2_ are clearly visible at the **K** point, while the inset on the right shows the angle integrated spectrum of the WS_2_/Au­(111) interface; ℏω = 21.2 eV, *T* = 6 K; (b) 3D rendering of the full emission cone/bands of WS_2_/Au­(111), ℏω = 21.2 eV, *T* = 6 K; (c) d*I*/d*V* spectrum acquired on monolayer WS_2_/Au­(111), showing both the band gap and interface states located at +1.1 eV, set point Δ*V* = 4 V, *I* = 30 pA, *T* = 77 K.

As a direct-to-indirect band gap transition is observed on bilayer formation,^1,10,11^ assessing the number of layers is crucial for understanding the band alignment at the interface. Our ARPES data taken with a photon energy of 21.2 eV show that the valence band maximum (VBM) at the ***K̅*** point (*E* – *E*_F_ = −1.3 eV) is higher (direct band gap) than at **Γ̅** (*E* – *E*_F_ = −1.65 eV), see Figure 3a, suggesting that we are in the monolayer regime.^46^ To exclude the presence of other electronic bands that could be suppressed by final states effects, we also acquired ARPES spectra at higher photon energy of 40.2 eV (as reported in Figure S2). These data, shown in Figure S2d, show the presence of an additional band at **Γ̅**, albeit extremely faint, which could be related to the presence of the second layer. Despite this evidence, no STM images with a clear presence of the second layer have been measured. Moreover, from the same data set, we observed extra bands at the ***M̅*** point, which are consistent with the formation of projected band gap edges induced by the interaction with the underlying Au(111) substrates.^47^

Figure 3c shows the d*I*/d*V* spectrum acquired on the pristine WS_2_/Au­(111) interface. In the negative bias region, where tunneling occurs from the occupied states of the sample, we identify the valence band edge located at about −1.3 V. This value matches the one obtained from the ARPES experiments. At positive sample bias instead, the differential conductance starts to increase at approximately +0.6 V and exhibits a broader maximum around +1.1 V. We also point out that the presence of a negative conductance at ∼+2.5 V is an artifact introduced by the low-noise transimpedance amplifier (TIA) of the STM caused by a steep and fast variation of the signal in that point. As discussed in Section S3 of the Supporting Information, we identify the feature at +1.1 V as a genuine electronic state rather than a tip-induced artifact. According to Eickholt et al.,^48^ at this energy lay two different states: the conduction-band minimum at the *K* point, and another state located at Γ that originates from the interaction between WS_2_ and Au. The lack of momentum resolution of STS does not allow us to further clarify the origin of this peak.

We therefore estimate the transport gap as the energy difference between the VBM at −1.3 eV and the onset of this feature, yielding a value of approximately 1.9 eV. This value is in line with previous STS and inverse photoemission studies on WS_2_/Au­(111) and related TMD/metal systems,^45–47^ but somewhat smaller than the quasiparticle gaps reported for freestanding WS_2_ monolayers^48,49^ (2.2–2.7 eV), which is readily understood in terms of band gap renormalization due to the strong dielectric screening of the metallic substrate.

This value is smaller than the 2.5 eV obtained from our *G*_0_ *W*_0_ calculation presented in the next section, which is performed for a freestanding WS_2_ layer. A fully quantitative description would require *GW* calculations for the complete WS_2_/Au­(111) heterostructure, which is currently beyond the scope of this work. We adopt this experimental estimate in the discussion of the energy-level alignment presented in the following sections.

We next address how this system is modified upon molecular adsorption of pentacene, focusing first on the geometrical properties of the adsorbate layer. The deposition of a single layer of pentacene molecules on pristine WS_2_ results in a self-assembled structure characterized by long-range ordering, as confirmed by the large-scale STM image shown in Figure 4a. Note that, at the chosen bias, the WS_2_/Au­(111) moiré pattern is still visible through the molecular layer. The self-assembled molecular structure is identified by high-resolution STM images presented in Figure 4b and by Fourier analysis of the large scale STM image in Figure 4c. Both reveal that pentacene forms an oblique unit cell with, respectively, 0.92 nm, Figure 4d, and 1.75 nm, Figure 4e, lattice constants, leading to a commensurate superstructure with respect to WS_2_ which can be described by the matrix $\left[\begin{array}{cc} 5 & -1\\ 0 & 3 \end{array}\right]$ , see Figure S4. Due to the hexagonal symmetry of WS_2_, 3 rotational domains are observed, as elucidated in Figure 4c. It is worth noting that the continuity of the grown WS_2_ monolayer above the one-atom deep Au depressions is also reflected by the molecular superstructure, which does not show evidence of accumulation points in the presence of the depression edges, as could be expected in the proximity of atomic steps.

**Figure 4:**
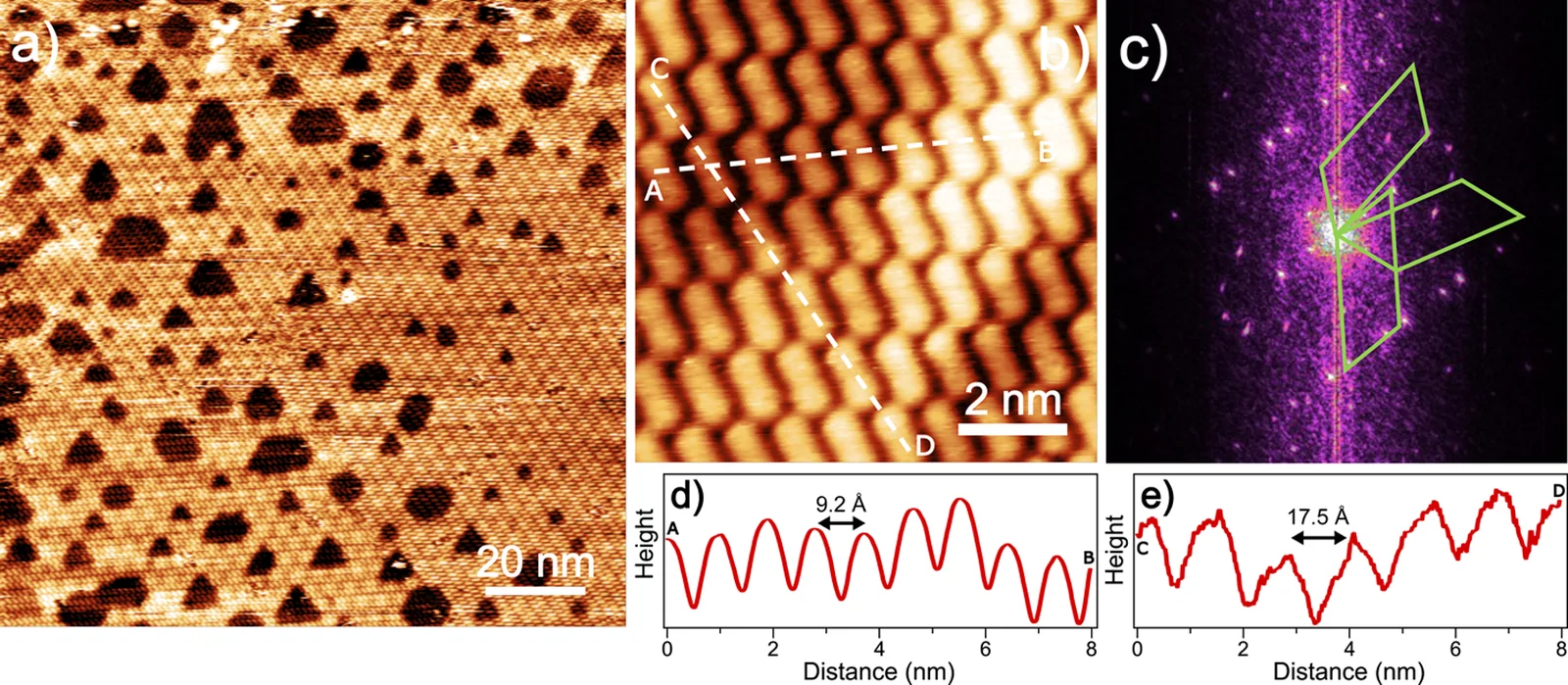
(a) Large-scale STM image of pentacene self-assembly on the WS_2_/Au­(111) interface, *V* = −1.4 V, *I* = 20 pA, (130 × 130) nm^2^, *T* = 77 K; (b) close up of pentacene molecular layer atop WS_2_ monolayer, *V* = −1.4 V, *I* = 20 pA, (8.5 × 8.5) nm^2^, *T* = 77 K; (c) fast Fourier transform of image (a) where the three different rotational domains of 5A on the hexagonal symmetry of WS_2_ are highlighted in green; (d) measured periodicity of pentacene self-assembled monolayer short axis; (e) measured periodicity of pentacene self-assembled monolayer long axis.

Having established the structural properties of the 5A/WS_2_ hybrid interface, we next focus on its electronic properties, which we investigated by means of STS. The corresponding spectra are shown in Figure 5a. The d*I*/d*V* curve acquired on the 5A/WS_2_ interface is superimposed on that acquired on pristine WS_2_/Au­(111) (in red).

**Figure 5:**
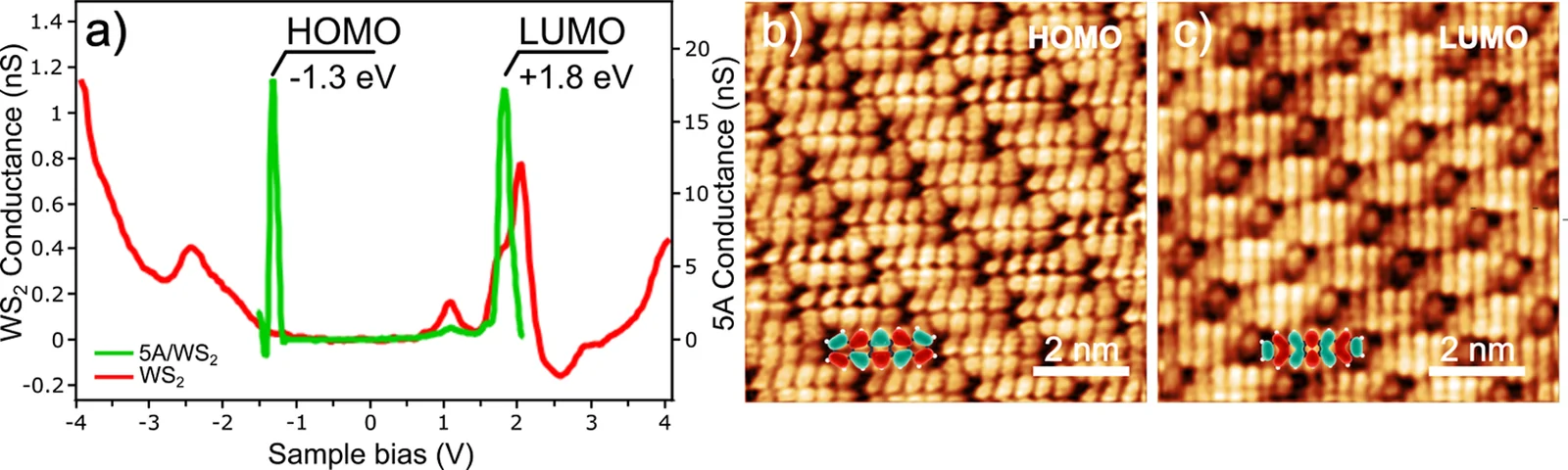
(a) d*I*/d*V* spectrum of pentacene monolayer atop WS_2_/Au­(111), green line, superimposed to the d*I*/d*V* spectrum of pristine WS_2_/Au­(111), red line, set point Δ*V* =1.5 V, *I* = 10 pA, *T* = 77 K; (b) STM image of 5A-HOMO molecular orbital, *V* = −1.3 V, *I* = 20 pA, (7 × 7) nm^2^, *T* = 77 K; (c) STM image of 5A-LUMO molecular orbital, *V* = +1.8 V, *I* = 20 pA, (7 × 7) nm^2^, *T* = 77 K.

The STS of the 5A/WS_2_ interface has two prominent peaks located at −1.3 eV and +1.8 eV. We were able to achieve orbital resolution at the corresponding bias voltages, Figure 5b,c, which can be unambiguously associated with the HOMO and LUMO, respectively,^50^ revealing a HOMO–LUMO gap of 3.1 eV. The magnitude of the pentacene gap also acts as an indication of its interaction with the underlying WS_2_ substrates: for gas-phase pentacene (noninteracting), the reported^50^ gap is 5.2 eV, while it shrinks to 2.2 eV for 5A on Au(111).^51^ In between this range, other reported 5A gaps on nonmetallic surfaces are 3.4 eV for 5A/h-BN/Rh(111)^52^ and 4.1 for 5A/NaCl/Cu(111).^53^

Concerning the electronic level alignment, we observe that the HOMO lies slightly above the VBM, i.e., within the electronic gap of WS_2_, while the LUMO is located within the WS_2_ conduction band. The reliability of our STS measurements is further supported by the persistence of the WS_2_ state at +1.1 eV in the spectra after pentacene deposition, albeit with reduced intensity. This indicates that charging effects did not artificially shift the HOMO–LUMO peak positions. Furthermore, it is worth noticing that the position of the interface states does not affect the overall band alignment of the hybrid interface as the pentacene LUMO is anyways superimposed to WS_2_ states, contrary to the HOMO.

Despite the small energy difference between the VBM and HOMO and the uncertainty in determining the exact VBM position, as discussed above, the energy level alignment suggests a type II band alignment.

In order to corroborate these experimental findings, we perform electronic structure calculations for the 5A/WS_2_ interface with the experimentally obtained geometry as a starting point, albeit without the underlying Au(111) substrate. We use van der Waals-corrected DFT to relax the geometry and obtain the structure shown in Figure 6a. Since the correct energy level alignment in such hybrid systems is far from trivial and DFT is known to typically underestimate the gap, we employ many-body perturbation theory at the *G*_0_ *W*_0_ level on top of the DFT results. While previous theoretical studies on similar systems^54,55^ predict the LUMO to lie just within the onset of the conduction band (and thus a type-II level alignment), our DFT calculations with comparable methodology would incorrectly predict a type-I level alignment, see Figure S5. This difference can be explained by the different unit cells considered: in our case, the unit cell is derived from the experiment, while the larger unit cells in previous theoretical studies result in a decreased interface dipole because of the larger substrate surface area per molecule. However, since it is a well-known fact that fundamental gaps of both TMDs and organic molecules are severely underestimated by DFT with semilocal exchange–correlation functionals, we perform additional calculations based on many-body perturbation theory within the *G*_0_ *W*_0_ approximation. Those energy-level corrections lead to a considerable improvement over our DFT results. A direct comparison between the obtained STS spectra (see Figure 5a) and the projected density of states from *G*_0_ *W*_0_ (Figure 6b) shows a fair agreement, both for the molecular and the WS_2_ features.

**Figure 6:**
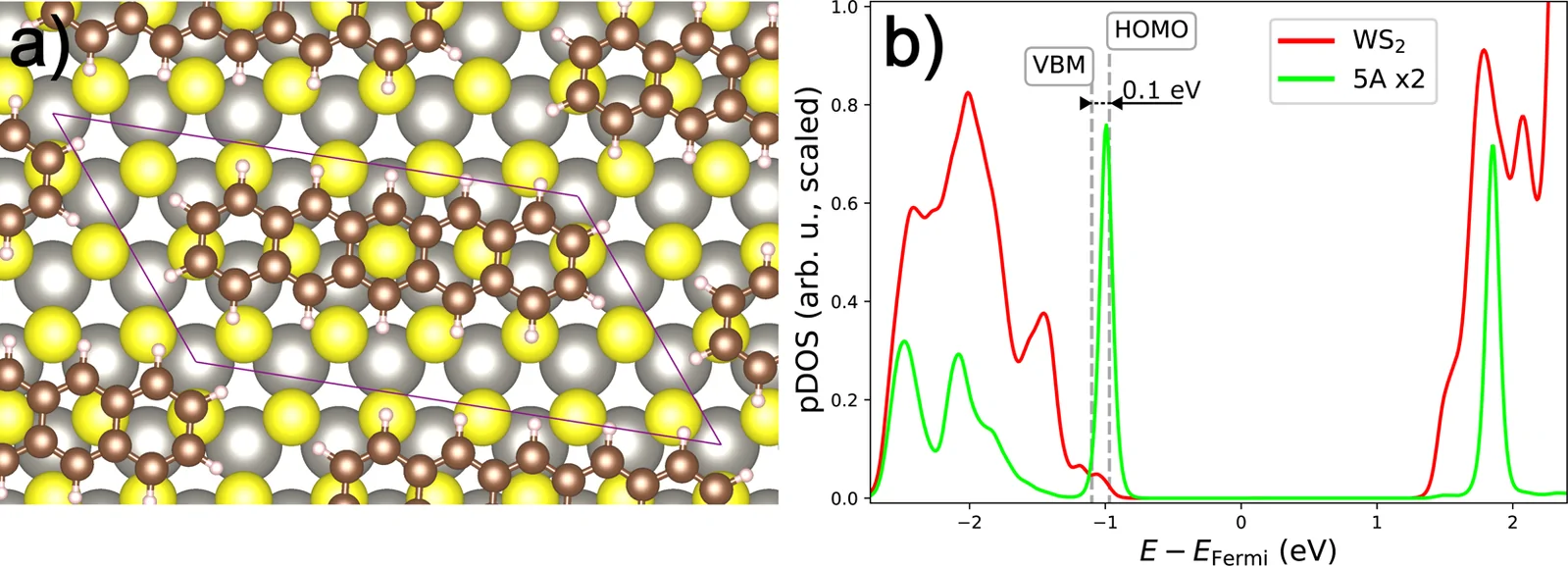
(a) Structure used in the DFT/*G*_0_ *W*_0_ calculations. W atoms are depicted in silver, S in yellow, C in brown, and H in white. The unit cell is indicated in purple. (b) Density of states from *G*_0_ *W*_0_ projected on WS_2_ (green) and on 5A (red, scaled by a factor of 2). Dashed lines marked with HOMO and VBM show the energy positions for the momentum maps depicted in Figure 7d,e, respectively.

On a quantitative level, the theoretical results deviate slightly from those of the experiment. For instance, we observe that the calculated HOMO–LUMO gap is somewhat smaller than in the experiment (2.8 eV vs 3.1 eV), which is a known effect of the starting point for *G*_0_ *W*_0_ calculations and could be mitigated by self-consistent *GW*. For a system of this size, the latter is computationally very demanding and beyond the scope of this work.

Given the challenges in both the computational and experimental approaches presented so far, a complementary technique is expedient in order to gain additional insight into the energy alignment, especially around the VBM. In this regard, photoemission orbital tomography^56^ enables the unambiguous identification of molecular orbitals of organic adsorbates and their energy positions, as well as the valence band signatures of WS_2_.

Within the POT approach, we measure the momentum distribution of the photoemitted electrons from a molecular film at a given kinetic energy and compare it with a cut of the 3D Fourier transform of a given molecular orbital. In order to identify the molecular resonances of interest at first, we compared the momentum-integrated photoemission spectra for the WS_2_/Au­(111) interface before and after the deposition of pentacene. The corresponding spectra are reported in Figure 7a. To better elucidate the changes induced by the molecular adlayer, we also show the difference between the two spectra. We determined the energy positions of the molecular features by fitting the molecular peaks with a Gaussian function.

**Figure 7:**
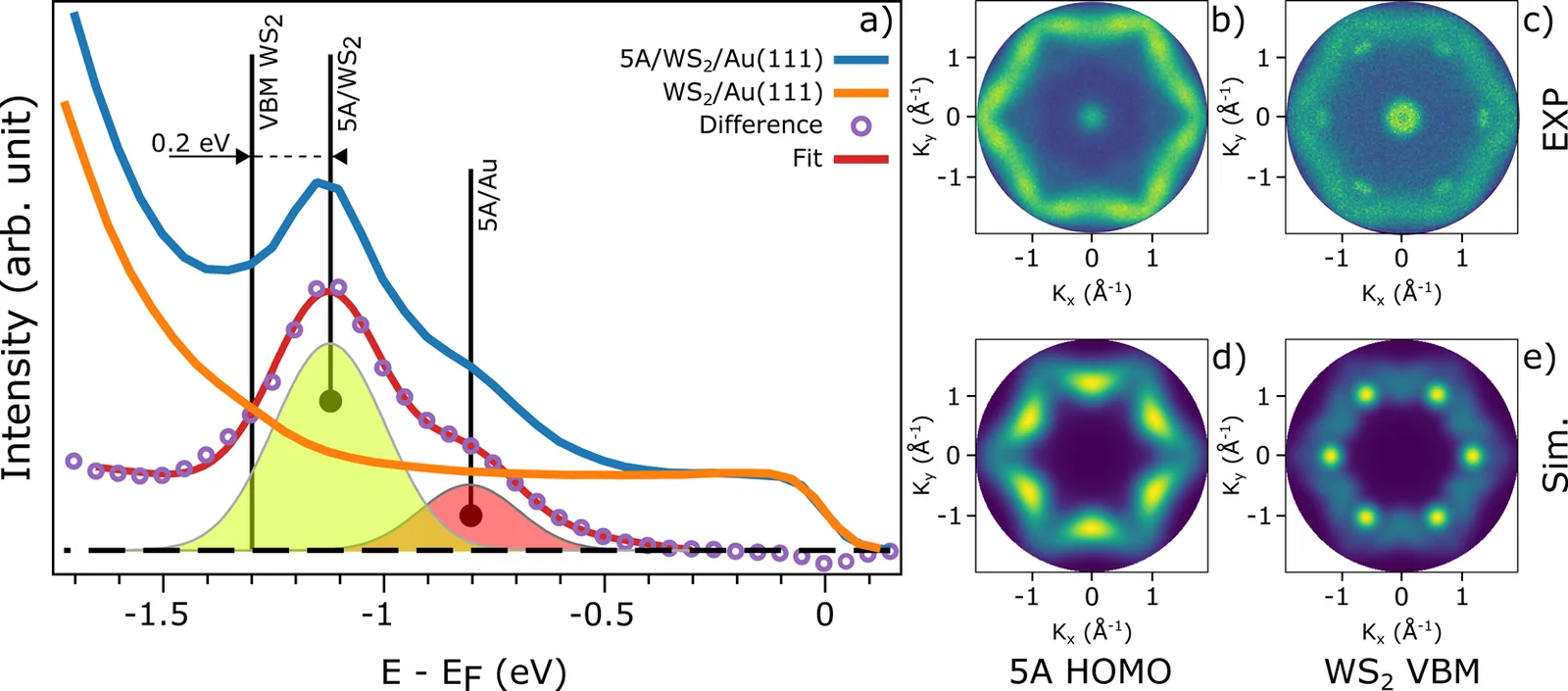
(a) Comparison of UPS spectra: pristine WS_2_/Au­(111), orange line, and pentacene monolayer, blue line. To enhance pentacene features, the difference between the UPS spectra, before and after 5A deposition, is plotted, red line; (b,c) symmetrized experimental pentacene POT averaged in the energy range associated only to 5A/WS_2_ interface and VBM of pristine WS_2_/Au­(111); (d,e) DFT-calculated 5A/WS_2_/Au­(111) POT and WS_2_/Au­(111) VBM. All spectrum and *k*-maps are acquired at ℏω = 26.4 eV, *T* = 77 K.

Upon molecular adsorption, two new peaks located at energies of −0.8 eV and −1.1 eV emerge. While the former (FWHM: 0.20 eV) is associated with 5A in direct contact with Au(111) (see also Figure S6), and is present because of the incomplete coverage (0.7 ML) of the Au(111) surface with WS_2_, the peak at −1.1 eV (fwhm: 0.17 eV) arises from 5A on WS_2_. The energy shift between the two pentacene species is due to the interaction with the different surface underneath. In order to assign the peak originating from 5A on WS_2_ to a specific 5A orbital, within the POT approach, we simulate photoemission momentum maps using a plane-wave final state^57^ and the DFT wave functions in conjunction with *G*_0_ *W*_0_ energy levels. This allows us to compare the experimental momentum map taken at the 5A HOMO energy position, Figure 7b, with the simulated map, Figure 7d. We would like to highlight that, since POT is a space-average technique, we need to take into account the different rotational domains of the substrate in order to obtain a meaningful comparison between theory and experiment. Therefore, we apply the corresponding symmetry operations to our simulated momentum maps. The overall procedure is explained in detail elsewhere.^58^ Our comparison between the simulated and measured momentum maps, Figure 7b,d, respectively, confirms that the peak at −1.1 eV is indeed the pentacene HOMO. The round feature at **Γ̅** originates from the WS_2_ second layer and becomes visible here because of the higher photon energy employed (namely 26.4 eV). It is worth noticing that even if the absolute values measured with different techniques are slightly different due to the different physical processes involved in each measurement, the relative positions between 5A HOMO and WS_2_ VBM are always consistent, i.e., the HOMO is always located at higher energy than WS_2_ VBM, thus proving the type-II energy level alignment.

We would also like to point out that the apparent energy degeneracy of the HOMO feature and the WS_2_ VBM in the calculations, as reported in the projected DOS presented in Figure 6b, is somehow relaxed when simulating momentum maps. This is because the spectral weight of the 6-fold features of the WS_2_ VBM is so low that the feature at this energy is dominated by emission from the HOMO level.

We now compare an experimental momentum map measured at an energy of −1.3 eV, 200 meV below the HOMO feature (Figure 7c), with the simulated map taken 100 meV below the HOMO feature in the calculated DOS (Figure 7e). These energies correspond to the onset of the VBM. Also in this case, we find good agreement between theory and experiment: the 6-fold features, which are characteristic of the WS_2_ VBM can be easily recognized in both the experimental and the simulated momentum maps. Another striking similarity between the two data sets is the presence of a faint background feature associated with the momentum-resolved HOMO signal. For the experimental data, this can be attributed to the large fwhm of the 5A HOMO emission, as shown in Figure 7a. Here, the left tail of the 5A HOMO peak is slightly superimposed to the WS_2_ VBM, located ∼0.2 eV below the pentacene feature, but we emphasize that the overall spectral weight is quite low. For our simulations, a similar argument holds.

Our overall conclusion is that the interface exhibits a type II energy level alignment, supported by STS data and further corroborated by POT and theoretical calculations.

## Conclusions

In summary, we have grown a novel hybrid organic–TMD interface, pentacene on WS_2_, and employed a complementary spectro-microscopy approach to investigate its structural and electronic properties. By means of molecular beam epitaxy (MBE) growth, we were able to obtain a high-quality, extended, and atomically flat WS_2_ film, using a novel precursor, namely DMDS, as a sulfur source.

The direct growth of TMD on metallic single-crystal substrates enables single-crystalline domains, but yields strong interfacial coupling, as observed by the presence of an interface hybrid state in STS spectra. Subsequent deposition of a single layer of pentacene results in an ordered commensurate superstructure on top of WS_2_, which is characterized by three distinct rotational domains and an oblique unit cell. STS spectra, supported by *G*_0_ *W*_0_ calculations, locate the pentacene LUMO within the conduction band of the WS_2_, while being indecisive in determining the precise relative energy position of the HOMO with respect to the VBM. This ambiguity has been resolved by our POT data, which revealed that the HOMO lies above the WS_2_ VBM, confirming the energy level alignment to be of a type II, even when considering the interface state in the conduction band.

This system represents therefore an ideal starting point to achieve a separation of electrons and holes in different layers. In more detail, we suggest that the possibility of intercalating a decoupling layer at the WS_2_/Au­(111) interface might be an effective method to prevent the quenching of excitonic resonances and to suppress the interface state at the same time. In fact, this particular energy level alignment, together with an extended and well-ordered interface, may favor the establishment of interlayer excitons between WS_2_ and pentacene, provided good decoupling from the substrate, making this interface a prototypical system for studying nonequilibrium phenomena with time-resolved-based techniques.

## Methods

### Experimental Section

The experiments have been performed in three different UHV chambers with a base pressure better than 2 × 10^–10^ mbar.

The Au(111) sample was cleaned by repeated cycles of sputtering with 2 keV Ar^+^ ions for 40 min, followed by annealing at 950 K for 30 min. The WS_2_ monolayer was grown by reactive MBE in a four steps process, evaporating tungsten in the dimethyl disulfide (DMDS) atmosphere. To favor the complete sulfurization of tungsten atoms, DMDS was injected in the UHV chamber exploiting a nozzle placed as close as possible to the Au(111) surface. First, the Au(111) substrate was exposed to DMDS at 950 K for 10 min, with the nozzle placed at 1 mm from the surface, with a chamber back-pressure *p*_DMDS_ = 2.5 × 10^–7^ mbar in order to deposit sulfur atoms on the surface before deposition of tungsten. The second step consisted in the deposition of tungsten in the DMDS atmosphere, chamber back-pressure of *p*_DMDS_ = 3.5 × 10^–7^ mbar with the nozzle placed at 5 mm from the surface, still holding the sample at 950 K. The evaporation rate was calibrated to achieve 1 ML of tungsten, equal to the atomic surface density of gold, in 15 min. The third step consisted in a postannealing of the grown WS_2_ in the DMDS atmosphere, using a chamber back-pressure *p*_DMDS_ = 2.5 × 10^–7^ mbar with the nozzle placed at 1 mm from the surface, at 950 K for 30 min. Finally, the fourth step consisted in a second postannealing without DMDS, lowering the temperature from 950 to 775 K, in order to stabilize the grown structure and desorb DMDS molecules that remained stuck to the surface during the growth. To avoid nucleation of WS_2_ into a second layer, we deposited a nominal coverage of 0.7 ML, resulting in the exposure of some clean gold surface.

The pentacene (5A) monolayer was deposited on as-grown WS_2_/Au­(111) by thermal evaporation from a commercial Knudsen cell (Kentax GmbH) at 450 K. The 5A evaporation rate was calibrated by means of a quartz microbalance.

X-ray photoelectron spectroscopy (XPS) was performed with a monochromatized Al Kα radiation source (SPECS μFocus 600) and an electron analyzer with a spectral energy resolution of 400 meV (SPECS PHOIBOS 150). XPS spectra were fitted using a pseudo-Voigt line shape, with Lorentz–Gauss weights of, respectively, 0.80–0.20, and the kinetic to binding energy conversion is given with respect to the Fermi edge of the Au(111) substrate.

Scanning Tunneling Microscopy and spectroscopy data were acquired using a CreaTec low-temperature STM, utilizing an electrochemically etched tungsten tip. All the reported images and spectra have been collected either at 77 or 4 K. For each STM image, the corresponding temperature is reported in the figure caption. Scanning tunnelling spectra were measured by first-derivative detection by means of an SRS lock-in amplifier: the bias voltage was modulated with a sinusoidal signal (amplitude 50 mV, frequency 2345 Hz, chosen to avoid any resonance with high harmonics of the power-line and other frequencies present in the system). The phase of the lock-in detection was determined by withdrawing the tip until no tunnel current was measured, so that only the capacitive component could flow, and selecting a value 90° smaller than the setting maximizing the capacitive signal. An integration time of 10 ms was chosen for the lock-in, and each spectrum was measured subsequently for an increasing and decreasing voltage ramp, lasting 30 s each, in order to detect any systematic effect. Depending on the noise level, 1–10 repeated measurements were averaged. Normalization of the conductance units was carried out by comparison with the numerically calculated derivative of the current, which leads to a noisier, yet correctly normalized curve.

ARPES measurements, momentum maps, and POT of WS_2_/Au­(111) and 5A/WS_2_/Au­(111) systems were acquired with a KREIOS 150 MM momentum microscope by SPECS GmbH. The instrument is coupled to two different light sources: a monochromatized helium discharge lamp (UVS300 and TMM150, from SPECS) and a high harmonic generation (HHG) chamber, generating photon energies of, respectively, 21.2 and 26.4 eV. The angle of incidence on the samples of both sources is 68° with respect to the surface normal. A detailed description of the HHG chamber can be found elsewhere.^59^ Measurements were acquired both at 77 and 6 K.

A 3D rendering of the full emission cone for the electronic band structure of WS_2_/Au­(111) is achieved using Blender equipped with the Microscopy odes^60^ add-on.

### Computational Approach

For our theory approach, we simulated 5A on a monolayer of WS_2_, ignoring the Au(111) substrate, since this would result in a prohibitively large unit cell due to the lattice mismatch between Au and WS_2_. We use a supercell of [[5 −1][0 3]] (in terms of the WS_2_ primitive cell with an in-plane lattice constant of 3.158 (Å)) and place the molecular center above a sulfur atom. For the geometric structure optimizations within DFT, we use the QuantumEspresso code^61–63^ with norm-conserving pseudopotentials from the PseudoDojo library^64,65^ and the vdw-df-ob86 functional to account for exchange–correlation effects and van der Waals^66^ interactions. The geometry relaxation was carried out until all forces were below 0.02 eV/Å, using a plane-wave cutoff of 80 Ry for the wave functions (320 Ry for the density) and a 2 × 4 × 1 shifted grid for the sampling of the Brillouin zone (BZ). To avoid spurious interaction between repeating slabs, we inserted a vacuum layer of 20 Å in conjunction with a truncation of the Coulombic interaction in the *z*-direction.^67^ Using the relaxed structure, we computed the charge density with the same settings as above, albeit with a 60 Ry cutoff for the plane waves, a *k*-mesh of 4 × 8 × 1, and the inclusion of spin–orbit interaction with a spinor representation of the wave functions. From the charge density, we compute wave functions and energies for the unoccupied states in a nonself-consistent run, using a **Γ̅**-centered 6 × 12 × 1 *k*-mesh. Using the energies and wave functions from the DFT calculation, we computed the many-body corrections to the energies on the *G*_0_ *W*_0_ level with the Yambo code.^68,69^ For the screened interaction *W*, we used a sum over 2400 bands and a cutoff of 10 Ry, and 2400 bands for *G* in conjunction with the Bruneval–Gonze method for accelerating convergence.^70^ We truncate the Coulombic interaction in the direction perpendicular to the slab^71^ and use a BZ interpolation for 2D systems.^72^

## Supplementary Material



## Notes

Additional STM data that confirm the continuity of WS_2_ across the atomically thin step edges, featuring ARPES data of the WS_2_/Au­(111) interface recorded at higher photon energy, alongside a comparison between pristine DFT and *G*_0_ *W*_0_ calculations; detailed STS-based discussion of the WS_2_/Au­(111) interface state at various sample positions; control experiment of pentacene deposited directly on Au(111); and in situ differential reflectance spectroscopy data tracked as a function of pentacene sublimation on WS_2_ (PDF)

## References

[1] Zeng M., Xiao Y., Liu J., Yang K., Fu L. (2018). Exploring
Two-Dimensional Materials toward the Next-Generation Circuits: From
Monomer Design to Assembly Control. Chem. Rev..

[2] Lv R. (2015). Transition Metal Dichalcogenides
and Beyond: Synthesis, Properties,
and Applications of Single- and Few-Layer Nanosheets. Acc. Chem. Res..

[3] Li P., Wen Y., He X., Zhang Q., Xia C., Yu Z. M., Yang S. A., Zhu Z., Alshareef H. N., Zhang X. X. (2017). Evidence for topological type-II
Weyl semimetal WTe_2_. Nat. Commun..

[4] Navarro-Moratalla E., Island J. O., Mañas-Valero S., Pinilla-Cienfuegos E., Castellanos-Gomez A., Quereda J., Rubio-Bollinger G., Chirolli L., Silva-Guillén J. A., Agraït N. (2016). Enhanced superconductivity in atomically thin TaS2. Nat. Commun..

[5] Rhodes D. A. (2021). Enhanced Superconductivity
in Monolayer *T*
_d_ -MoTe _2_. Nano Lett..

[6] Wang Y. D., Yao W. L., Xin Z. M., Han T. T., Wang Z. G., Chen L., Cai C., Li Y., Zhang Y. (2020). Band insulator
to Mott insulator transition in 1T-TaS2. Nat.
Commun..

[7] Mak K. F., He K., Shan J., Heinz T. F. (2012). Control of valley polarization in
monolayer MoS2 by optical helicity. Nat. Nanotechnol..

[8] Xia J., Yan J., Shen Z. X. (2017). Transition metal dichalcogenides:
structural, optical
and electronic property tuning via thickness and stacking. FlatChem.

[9] Yao X., Wang Y., Lang X., Zhu Y., Jiang Q. (2019). Thickness-dependent
bandgap of transition metal dichalcogenides dominated by interlayer
van der Waals interaction. Phys. E.

[10] Mak K. F., Lee C., Hone J., Shan J., Heinz T. F. (2010). Atomically Thin
MoS2: A New Direct-Gap Semiconductor. Phys.
Rev. Lett..

[11] Gehlmann M. (2017). Direct Observation of
the Band Gap Transition in Atomically Thin
ReS _2_. Nano Lett..

[12] Gong C., Zhang Y., Chen W., Chu J., Lei T., Pu J., Dai L., Wu C., Cheng Y., Zhai T. (2017). Electronic and Optoelectronic Applications Based on
2D Novel Anisotropic
Transition Metal Dichalcogenides. Adv. Sci..

[13] Li Q., Alfrey A., Hu J., Lydick N., Paik E., Liu B., Sun H., Lu Y., Wang R., Forrest S. (2023). Macroscopic transition metal dichalcogenides monolayers with uniformly
high optical quality. Nat. Commun..

[14] Xia F., Wang H., Xiao D., Dubey M., Ramasubramaniam A. (2014). Two-dimensional
material nanophotonics. Nat. Photonics.

[15] Waldrop M. M. (2016). The chips
are down for Moore’s law. Nature.

[16] Aftab S., Hegazy H. H. (2023). Emerging Trends in 2D TMDs Photodetectors and Piezo-Phototronic
Devices. Small.

[17] Mak K. F., Shan J. (2016). Photonics and optoelectronics
of 2D semiconductor transition metal
dichalcogenides. Nat. Photonics.

[18] Nassiri
Nazif K., Nitta F. U., Daus A., Saraswat K. C., Pop E. (2023). Efficiency limit of transition metal dichalcogenide solar cells. Commun. Phys..

[19] Sebastian A., Pendurthi R., Choudhury T. H., Redwing J. M., Das S. (2021). Benchmarking
monolayer MoS2 and WS2 field-effect transistors. Nat. Commun..

[20] Fang N., Chang Y. R., Fujii S., Yamashita D., Maruyama M., Gao Y., Fong C. F., Kozawa D., Otsuka K., Nagashio K. (2024). Room-temperature
quantum
emission from interface excitons in mixed-dimensional heterostructures. Nat. Commun..

[21] Li H. (2024). Imaging moiré
excited states with photocurrent tunnelling
microscopy. Nat. Mater..

[22] Rani S. (2022). Two-dimensional transition metal dichalcogenides and
their heterostructures:
Role of process parameters in top-down and bottom-up synthesis approaches. Mater. Sci. Semicond. Process..

[23] Kang T. (2022). Strategies for Controlled
Growth of Transition Metal Dichalcogenides
by Chemical Vapor Deposition for Integrated Electronics. ACS Mater. Au.

[24] Walsh, L. A. ; Addou, R. ; Wallace, R. M. ; Hinkle, C. L. Molecular Beam Epitaxy of Transition Metal Dichalcogenides. In Molecular Beam Epitaxy; Elsevier, 2018; pp 515–531.

[25] Nitschke J. E. (2025). Electronic and structural
coupling of pentacene on NiO(001). Nanoscale.

[26] Capra M., Fratesi G., Motti F., Picone A., Ferretti A., Milanesi P., Giampietri A., Goto F., Calloni A., Ciccacci F. (2025). Growth and Characterization of a CoTPP/NiO(001)
Antiferromagnetic Spinterface. Adv. Phys. Res..

[27] Capra M., Marino M., Picone A., Ferretti A., Giampietri A., Ciccacci F., Fiori S., Dagur D., Motti F., Vinai G. (2025). Long-range magnetic
ordering of FePc molecules driven
by interfacial coupling with antiferromagnetic Cr2O3. Phys. Rev. Mater..

[28] Otero R., Gallego J. M., de Parga A. L. V., Martín N., Miranda R. (2011). Molecular Self-Assembly
at Solid Surfaces. Adv. Mater..

[29] Thompson J. J. P., Gerhard M., Witte G., Malic E. (2023). Optical signatures
of Förster-induced energy transfer in organic/TMD heterostructures. npj 2D Mater. Appl..

[30] Bennecke W. (2025). Hybrid Frenkel–Wannier
excitons facilitate ultrafast energy
transfer at a 2D–organic interface. Nat.
Phys..

[31] Benini M., Parlak U., Bork S., Strohsack J., Leven R., Gutnikov D., Mertens F., Zhukov E., Rakshit R. K., Bergenti I. (2025). Light-driven
modulation
of proximity-enhanced functionalities in hybrid nano-scale systems. Nat. Commun..

[32] Strohsack J., Shumilin A. V., Zhao H., Jecl G., Kabanov V. V., Benini M., Rakshit R. K., Dediu V. A., Rogers M. D., Ozdemir S. (2025). Dynamics
of proximity-induced magnetism at cobalt/molecular
interfaces. Sci. Adv..

[33] Deng S., Li L., Li M. (2018). Stability of direct band gap under mechanical strains
for monolayer MoS2, MoSe2, WS2 and WSe2. Phys.
E.

[34] Longo R. C. (2017). Intrinsic air stability
mechanisms of two-dimensional transition
metal dichalcogenide surfaces: basal versus edge oxidation. 2D Mater..

[35] Ewert M., Buß L., Braud N., Kundu A. K., Sheverdyaeva P. M., Moras P., Genuzio F., Menteş T. O., Locatelli A., Falta J. (2021). The Transition
From
MoS2 Single-Layer to Bilayer Growth on the Au(111) Surface. Front. Phys..

[36] Stan R.-M. (2019). Epitaxial single-layer
NbS2 on Au(111): Synthesis, structure, and
electronic properties. Phys. Rev. Mater..

[37] Arnold F. (2018). Novel single-layer vanadium
sulphide phases. 2D Mater..

[38] Tyagi S., Lee K.-J., Shukla P., Chae J.-C. (2020). Dimethyl disulfide
exerts antifungal activity against Sclerotinia minor by damaging its
membrane and induces systemic resistance in host plants. Sci. Rep..

[39] Wang X. (2021). Effects of soil type,
moisture content and organic amendment rate
on dimethyl disulfide distribution and persistency in soil. Environ. Pollut..

[40] Bignardi L. (2019). Growth and structure
of singly oriented single-layer tungsten disulfide
on Au(111). Phys. Rev. Mater..

[41] Spurgeon P. M., Lai K. C., Han Y., Evans J. W., Thiel P. A. (2020). Fundamentals
of Au(111) Surface Dynamics: Coarsening of Two-Dimensional Au Islands. J. Phys. Chem. C.

[42] Biener M. M., Biener J., Friend C. M. (2007). Sulfur-induced mobilization of Au
surface atoms on Au(111) studied by real-time STM. Surf. Sci..

[43] Wang J. (2024). Epitaxial Growth of
Monolayer WS _2_ Single Crystals on
Au(111) Toward Direct Surface-Enhanced Raman Spectroscopy Detection. ACS Nano.

[44] Kastl C. (2018). Multimodal spectromicroscopy of monolayer WS _2_ enabled
by ultra-clean van der Waals epitaxy. 2D Mater..

[45] Katoch J. (2018). Giant spin-splitting and gap renormalization driven
by trions in
single-layer WS2/h-BN heterostructures. Nat.
Phys..

[46] Dendzik M. (2015). Growth and electronic
structure of epitaxial single-layer WS2 on
Au(111). Phys. Rev. B: Condens. Matter Mater.
Phys..

[47] Bruix A. (2016). Single-layer MoS2 on
Au(111): Band gap renormalization and substrate
interaction. Phys. Rev. B.

[48] Eickholt P. (2018). Spin Structure of K Valleys in Single-Layer WS2 on Au(111). Phys. Rev. Lett..

[49] Krane N., Lotze C., Franke K. J. (2018). Moiré
structure of MoS2 on
Au(111): Local structural and electronic properties. Surf. Sci..

[50] Duan S., Tian G., Xu X. A. (2023). General
Framework of Scanning Tunneling
Microscopy Based on Bardeen’s Approximation for Isolated Molecules. JACS Au.

[51] Smerdon J. A., Bode M., Guisinger N. P., Guest J. R. (2011). Monolayer and bilayer
pentacene on Cu(111). Phys. Rev. B: Condens.
Matter Mater. Phys..

[52] Koslowski S. (2017). Adsorption and electronic
properties of pentacene on thin dielectric
decoupling layers. Beilstein J. Nanotechnol..

[53] Repp J., Meyer G., Stojković S. M., Gourdon A., Joachim C. (2005). Molecules
on Insulating Films: Scanning-Tunneling Microscopy Imaging of Individual
Molecular Orbitals. Phys. Rev. Lett..

[54] Black E., Kratzer P., Morbec J. M. (2023). Interaction between
pentacene molecules
and monolayer transition metal dichalcogenides. Phys. Chem. Chem. Phys..

[55] Black E., Morbec J. M. (2024). Effect of molecular
rotation and concentration on the
adsorption of pentacene molecules on two-dimensional monolayer transition
metal dichalcogenides. Electron. Struct..

[56] Zamborlini G., Lüftner D., Feng Z., Kollmann B., Puschnig P., Dri C., Panighel M., Di Santo G., Goldoni A., Comelli G. (2017). Multi-orbital charge
transfer at highly oriented organic/metal interfaces. Nat. Commun..

[57] Lüftner D. (2017). Understanding the photoemission
distribution of strongly interacting
two-dimensional overlayers. Phys. Rev. B.

[58] Janas D. M. (2026). Correlation-Driven Band Modifications Promote
Chemical Bonding at
3 Ferromagnetic Surfaces. Small.

[59] Schiller K. J., Sternemann L., Stupar M., Omar A., Hoffmann M., Nitschke J. E., Mischke V., Janas D. M., Ponzoni S., Zamborlini G. (2025). Time-resolved momentum microscopy with fs-XUV
photons at high repetition rates with flexible energy and time resolution. Sci. Rep..

[60] Gros O., Bhickta C., Lokaj G., Schwab Y., Köhler S., Banterle N. (2025). Microscopy Nodes: versatile
3D microscopy visualization
with Blender. bioRxiv.

[61] Giannozzi P., Baseggio O., Bonfà P., Brunato D., Car R., Carnimeo I., Cavazzoni C., de Gironcoli S., Delugas P., Ferrari Ruffino F. (2020). Quantum ESPRESSO toward
the exascale. J. Chem. Phys..

[62] Giannozzi P. (2017). Advanced
capabilities for materials modelling with Quantum ESPRESSO. J. Phys.: Condens. Matter.

[63] Giannozzi P. (2009). QUANTUM ESPRESSO: a
modular and open-source software project for
quantum simulations of materials. J. Phys.:
Condens. Matter.

[64] van
Setten M. J. (2018). The PseudoDojo: Training and grading a 85 element
optimized norm-conserving pseudopotential table. Comput. Phys. Commun..

[65] Hamann D. R. (2013). Optimized
norm-conserving Vanderbilt pseudopotentials. Phys. Rev. B: Condens. Matter Mater. Phys..

[66] Klimeš J., Bowler D. R., Michaelides A. (2011). Van der Waals density functionals
applied to solids. Phys. Rev. B: Condens. Matter
Mater. Phys..

[67] Sohier T., Calandra M., Mauri F. (2017). Density functional
perturbation theory
for gated two-dimensional heterostructures: Theoretical developments
and application to flexural phonons in graphene. Phys. Rev. B.

[68] Sangalli D. (2019). Many-body perturbation
theory calculations using the yambo code. J.
Phys.: Condens. Matter.

[69] Marini A., Hogan C., Grüning M., Varsano D. (2009). yambo: An ab initio
tool for excited state calculations. Comput.
Phys. Commun..

[70] Bruneval F., Gonze X. (2008). Accurate GW self-energies
in a plane-wave basis using only a few
empty states: Towards large systems. Phys. Rev.
B: Condens. Matter Mater. Phys..

[71] Rozzi C. A., Varsano D., Marini A., Gross E. K. U., Rubio A. (2006). Exact Coulomb
cutoff technique for supercell calculations. Phys. Rev. B: Condens. Matter Mater. Phys..

[72] Guandalini A., D’Amico P., Ferretti A., Varsano D. (2023). Efficient GW calculations
in two dimensional materials through a stochastic integration of the
screened potential. npj Comput. Mater..

