# Supplementary material for: Bottom-Up Realization of a Type-II Organic/Transition-Metal Dichalcogenides Heterointerface: Pentacene on Monolayer WS2
Source: ACS Nano. 2026 Sep 4;20(37):25410–9. doi: 10.1021/acsnano.6c07654 (PMC13602994; doi:10.1021/acsnano.6c07654)
Supplement: Supplementary file 1 [file nn6c07654_si_001.pdf]

SUPPORTING INFORMATION

Bottom-up realization of a type-II organic–Transition Metal Dichalcogenides heterointerface: Pentacene on monolayer WS_2

Michele Capra[1,*], Christian S. Kern[2], Mira S. Arndt[1], Karl J. Schiller[1], Max Niederreiter[2], Francesco Presel[2], Iolanda Di Bernardo[5], Marco Gruenewald[3], Torsten Fritz[3], Stefan Tappertzhofen[4], Martin Sterrer[2], Peter Puschnig[2], Mirko Cinchetti[1] and Giovanni Zamborlini[1,2,*].

[1] TU Dortmund University, Department of Physics, 44227 Dortmund, Germany

[2] Institute of Physics, NAWI Graz, University of Graz, 8010 Graz, Austria

[3] Institute of Solid State Physics, Friedrich Schiller University Jena, 07743 Jena, Germany

[4] TU Dortmund University, Department of Electrical Engineering and Information Technology, 44227 Dortmund, Germany

[5] Universidad Autónoma de Madrid and IFIMAC, 28049 Madrid, Spain

*corresponding authors: michele.capra@tu-dortmund.de; giovanni.zamborlini@uni-graz.at

1 – WS_2 continuity across atomically thin deeps

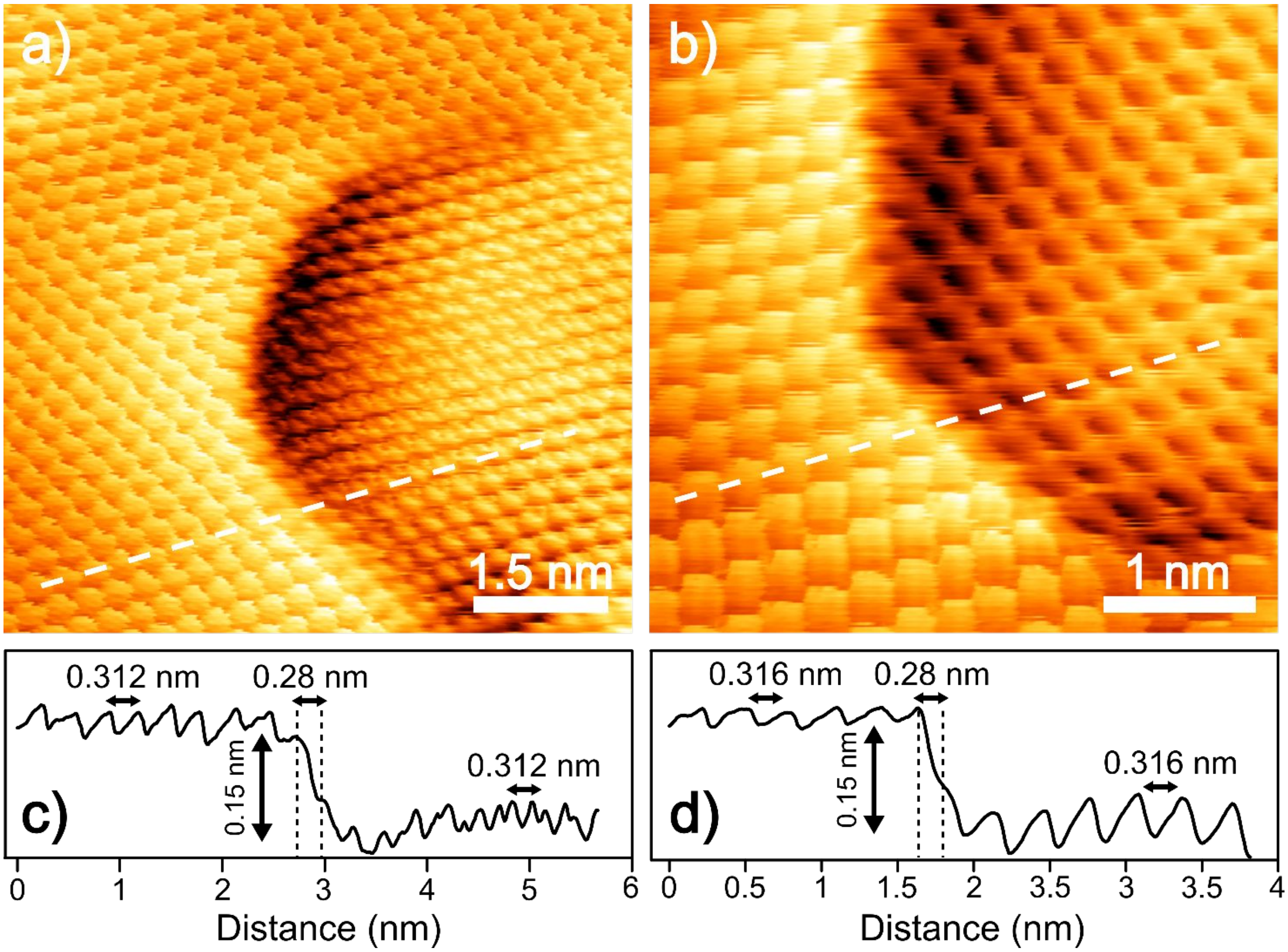

Figure S1 – Atomically resolved STM images of WS_2/Au(111) along atomic deeps; a) $V = -1.7\ V, I = 100\ pA, (7 \times 7)\ nm^2, T = 3.5\ K$; b) $V = -1.7\ V, I = 100\ pA, (4.13 \times 4.13)\ nm^2, T = 3.5\ K$; c) – d) Line profiles along step edge of atomically deeps in Au(111) showing that the atomic periodicity inside and outside the deeps is presence also on the atomic step, corroborating the WS_2 film continuity.

The STM images in Figure S1 have been acquired ex-situ, after exposing the sample to air and degassing it afterward in UHV at 350 °C, which did not show any sign of degradation.

Fig. S1 shows two different atomically resolved STM images of WS_2 along atomically thin deeps. Looking at the line profiles along the borders, Fig. S1 c) – d), it is clearly possible to observe the presence of a periodicity superimposed to the atomic step, elucidating the continuity of the WS_2 film without any interruption. Along the border we measured a slightly shorter lattice constant of 0.28 nm, compared to the surrounding regions, which might be related to a strain induced by the edge step. It is also possible to notice that the imaging contrast of the lattice is different inside and outside the deep, with an additional shorter periodicity inside the deep in Fig. S1a), which might hint to a different density of states/atomic arrangement of WS_2 with respect to Au(111) with respect inside and outside the deeps.

2 – WS_2 bilayer and projected band gap edges induce by Au(111)

Depending on the used photon energy, matrix element effects can play a crucial role in the observed electronic band structure of a material, highlighting or hindering key features. In particular, at 21.2 eV (HeI), any additional bands located at $\bar{\boldsymbol{\Gamma}}$ due to multilayer

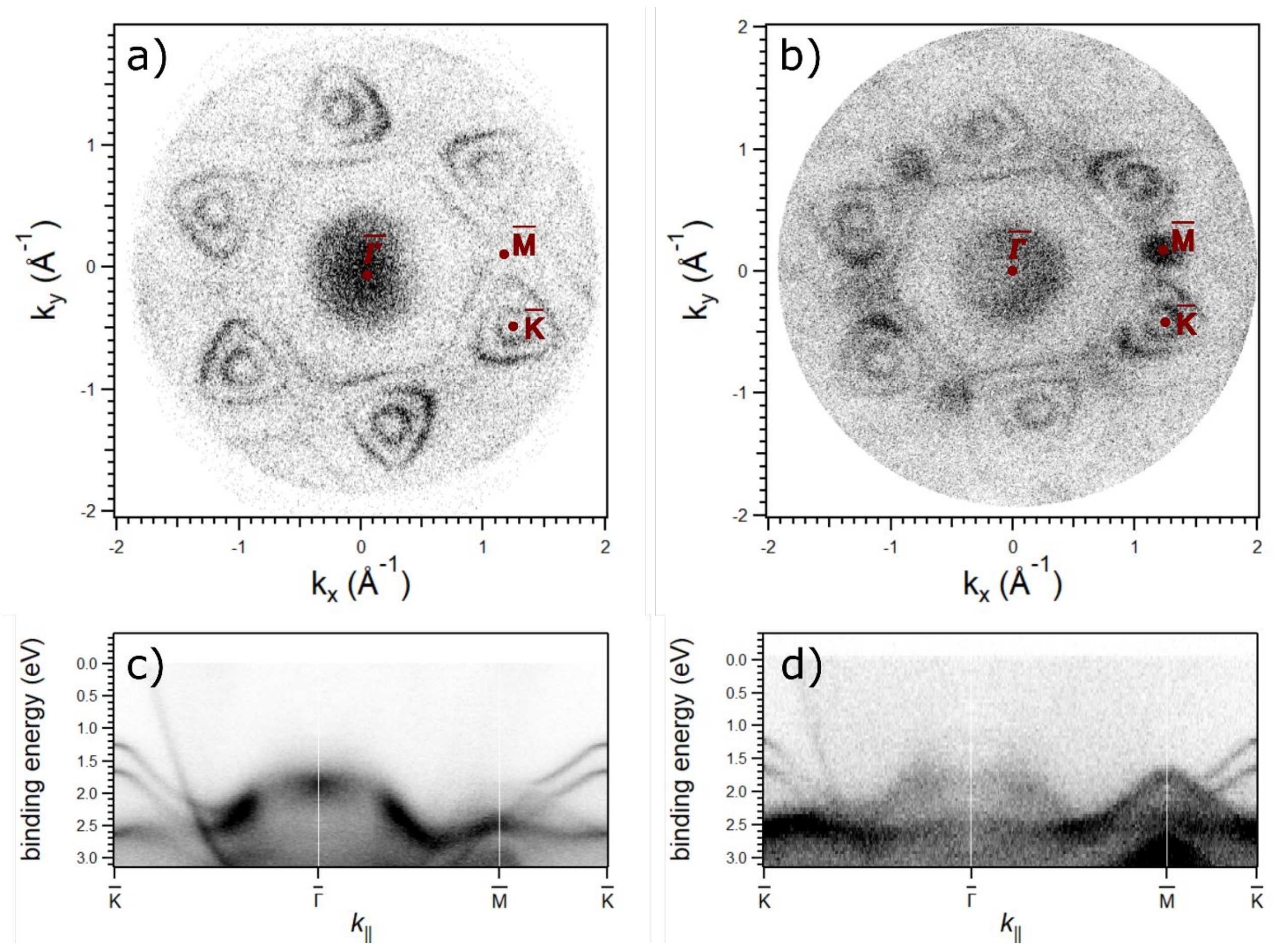

Figure S2 – a) Momentum map of WS_2/Au(111) acquired at E_b = 1.8 eV using $\hbar\omega = 21.2\ eV\ (HeI)$, $T = 6\ K$; b) Momentum map of WS_2/Au(111) acquired at E_b = 1.8 eV using $\hbar\omega = 40.8\ eV$ (HeII), $T = 6\ K$; c) Cut along high symmetry direction of a); d) Cut along high symmetry direction of b).

WS_2 are completely suppressed, Fig. S2a) and c). At 40.8 eV, HeII, contribution of those bands became stronger, allowing their identification, Fig. S2b) and d). Despite a lower signal-to-noise ratio, in Fig. S2d) it is possible to observe a really faint signal in $\bar{\boldsymbol{\Gamma}}$ related to some second layer enucleation.

It is worth mentioning that the interaction with the underlying Au(111) substrate might induce the formation of projected band gap edges. In particular, we observe in Fig. S2b and d) the presence of additional features located at the high symmetry point $\bar{\boldsymbol{M}}$, consistent with previous work and calculations on similar systems.

3 – Consistency of WS_2/Au(111) interface state

To further corroborate the presence of the feature located at +1 eV, we acquired different STS spectra at different positions and samples. Fig. S3 shows two examples where the same feature is clearly present, with different intensities, depending on the used set points. In Fig. S3b) the negative conductance at 2.5 eV is still present, which is an artifact of the STM current amplifier whenever there are steep variations of the signal.

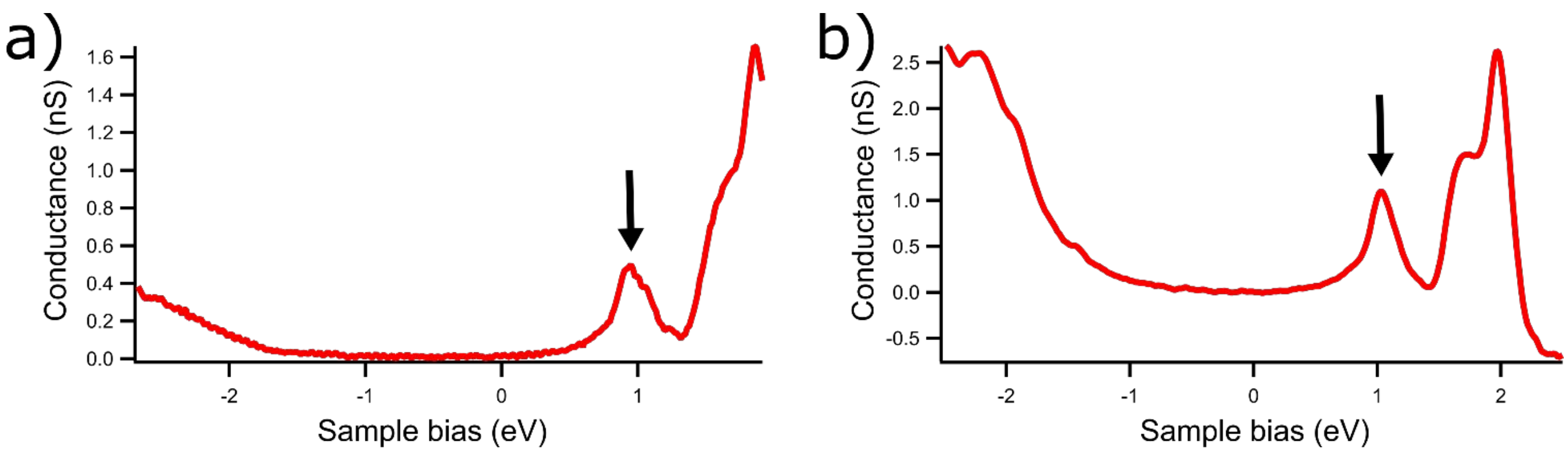

Figure S3 – Two examples of dI/dV spectra acquired on monolayer WS_2/Au(111) for different set points and samples, showing the same interface states located at +1eV, a) set point $\Delta V = 2\,V, I = 50\,pA, T = 77\,K$, b) $\Delta V = 2.5\,V, I = 30\,pA, T = 77\,K$.

4 – 5A/WS_2 unit cell

In Fig. S4 is shown the unit cell corresponding to the matrix $\begin{bmatrix} 5 & -1 \\ 0 & 3 \end{bmatrix}$. It is worth noticing that we are using the hexagonal lattice of WS_2 as a reference.

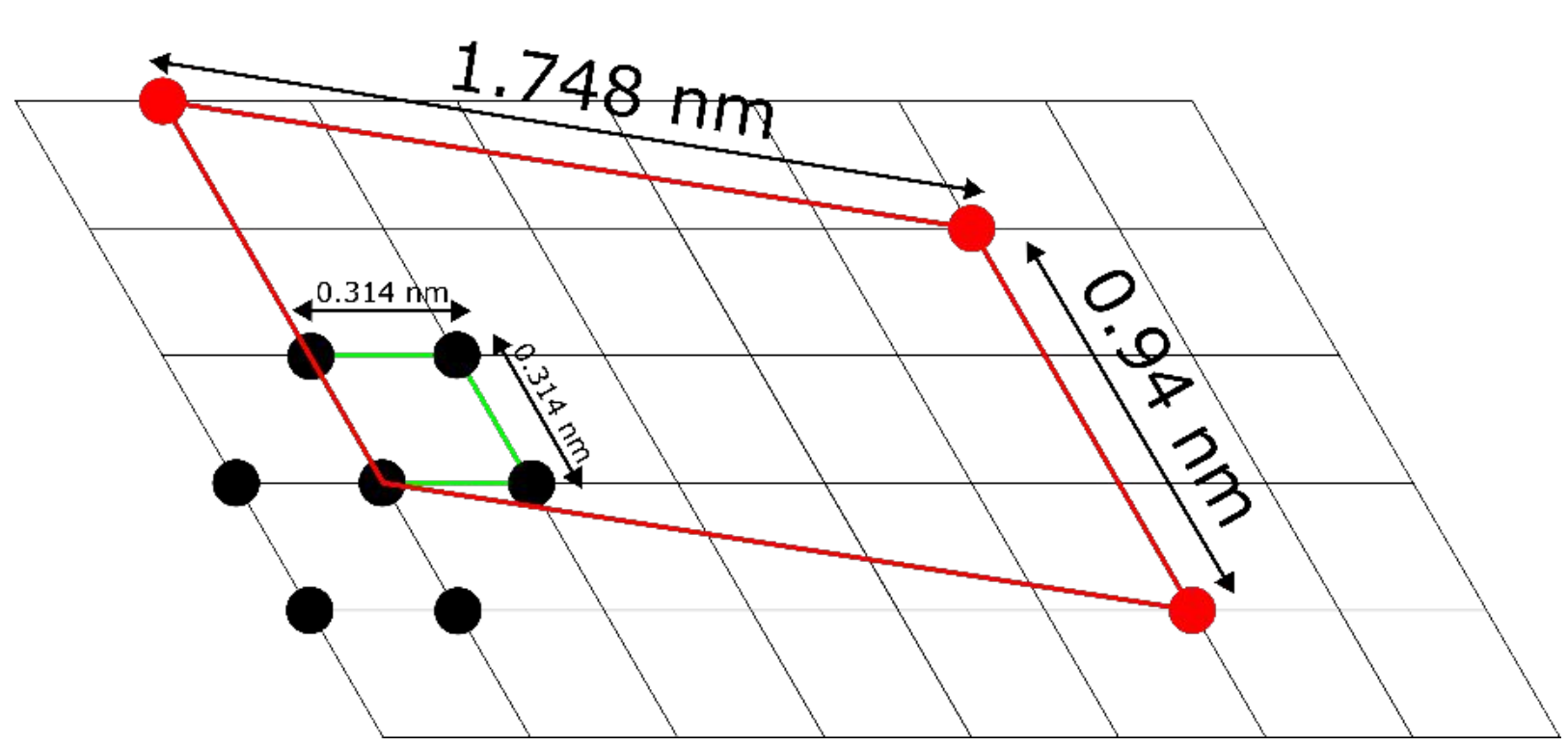

Figure S4 – Sketch of Pentacene supercell superimposed to WS_2 unit cell. Black points refer to WS_2 as guide.

5 – Comparison between pristine DFT and G_0W_0 calculations

In Fig. S5 is shown the comparison of pristine DFT and G_0W_0 level corrected calculations. As it is possible to observe in Fig. S4a), the relative positions of both pentacene HOMO

and LUMO are in disagreement with the experimental results. Moreover the predicted band alignment would have been type-I. Differences with respect to previous theoretical works on similar systems[1,2] arise from the different molecular geometry used for the calculations, which, in our case, is directly derived from experimental STM images. After considering G_0W_0 level corrections, Fig. S5b), both theory and experiments show good agreement in determining the band-alignment of the hybrid $5A/WS_2$ interface.

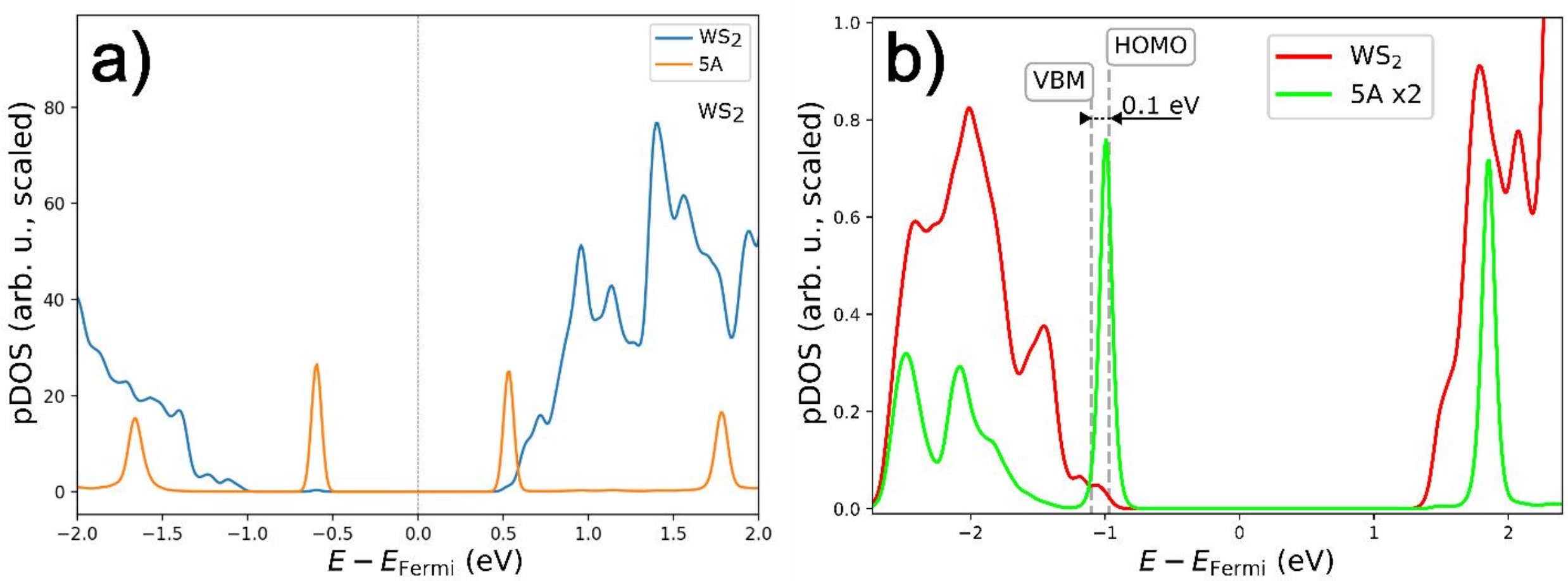

Figure S5 – a) Pristine DFT calculations of $5A/WS_2$ interface; b) G_0W_0 corrected DFT calculations

6 – Pentacene on clean Au(111)

Fig. S4 shows a comparison between the UPS spectra acquired on clean Au(111) and the 5A/Au(111) interface. Contrary to the $5A/WS_2$/Au(111) interface, now only one peak it is present, located at -0.85 eV, consistent with the smaller peak in Fig. 7. We thus conclude that the smaller peak located at around -0.8 eV on the $5A/WS_2$/Au(111) sample is caused by pentacene on clean Au(111) due to a non-perfect coverage of the gold surface by the WS_2.

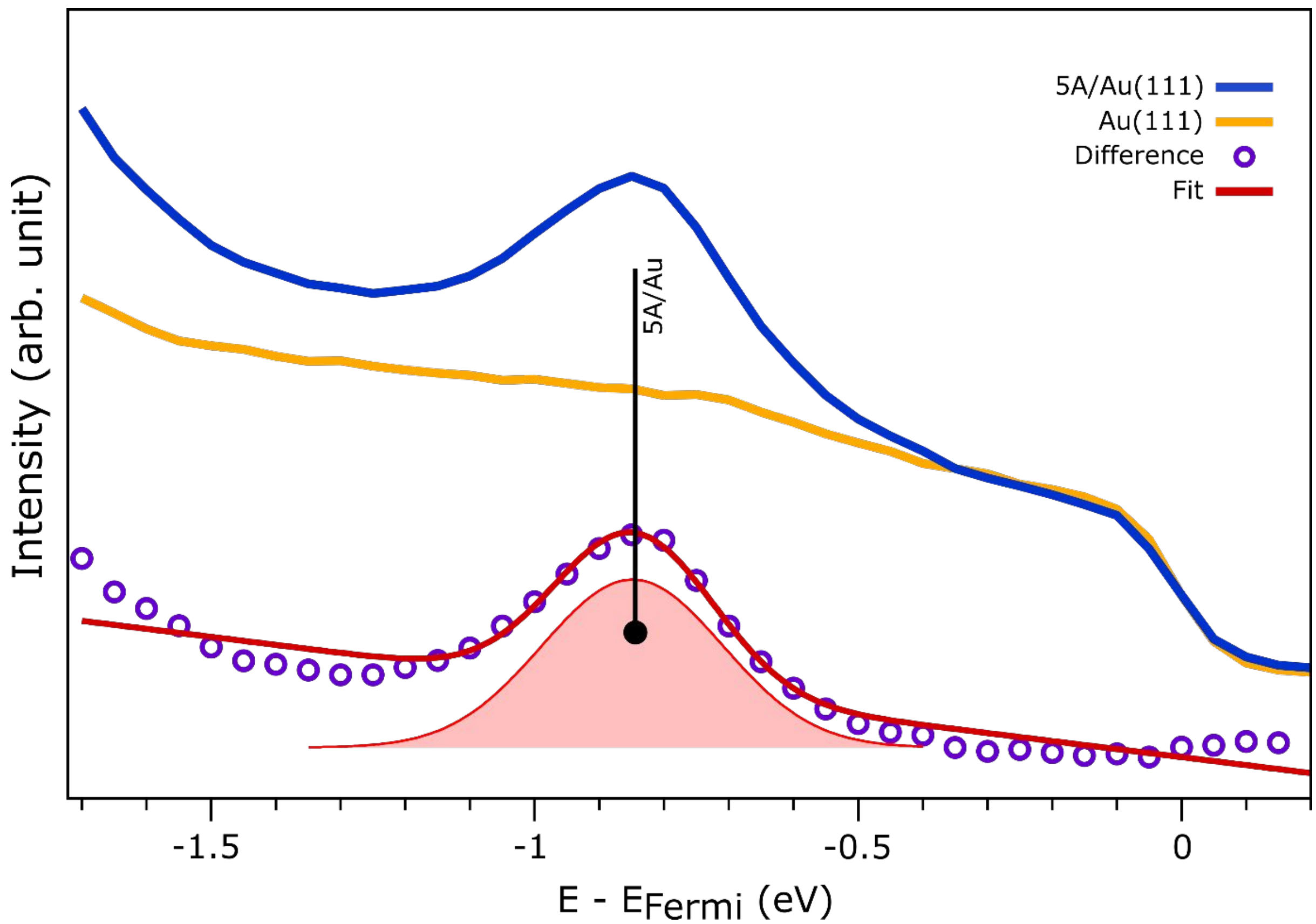

Figure S6 - Comparison of UPS spectra: pristine Au(111), yellow line, and pentacene monolayer atop clean Au(111), blue line. To enhance pentacene features, the difference between the UPS spectra, before and after 5A deposition, is plotted, violet dots. The difference spectrum was fitted with a gaussian superimposed to a linear background. All spectra are acquired at $\hbar\omega = 21.2\ eV, T = 300\ K$.

7 – Optical absorption of pentacene on WS_2/Au(111)

In order to characterize the optical absorption behavior of 5A on WS_2/Au(111) we performed in-situ differential reflectance spectroscopy (DRS). The DRS signal is defined as the relative change of the reflectivity R of the sample during our experiment, $(R - R_0)/R_0$, with R_0 being the reflectivity of the pristine substrate[3]. We used randomly polarized light under an angle of incidence of 20° with respect to the surface normal. A combination of a halogen lamp and broadband LEDs ("MBB1D1" from Thorlabs GmbH and "blue UVA-B LED E635" from Dr. Licht GmbH) were used as light sources, which we coupled together by a dichroic mirror ("DMLP490R" from Thorlabs GmbH). Further details about our optical setup can be found elsewhere. [1] The DRS measurement performed during the deposition of pentacene in a UHV chamber with a base pressure of $5 \cdot 10^{-10}$ mbar.

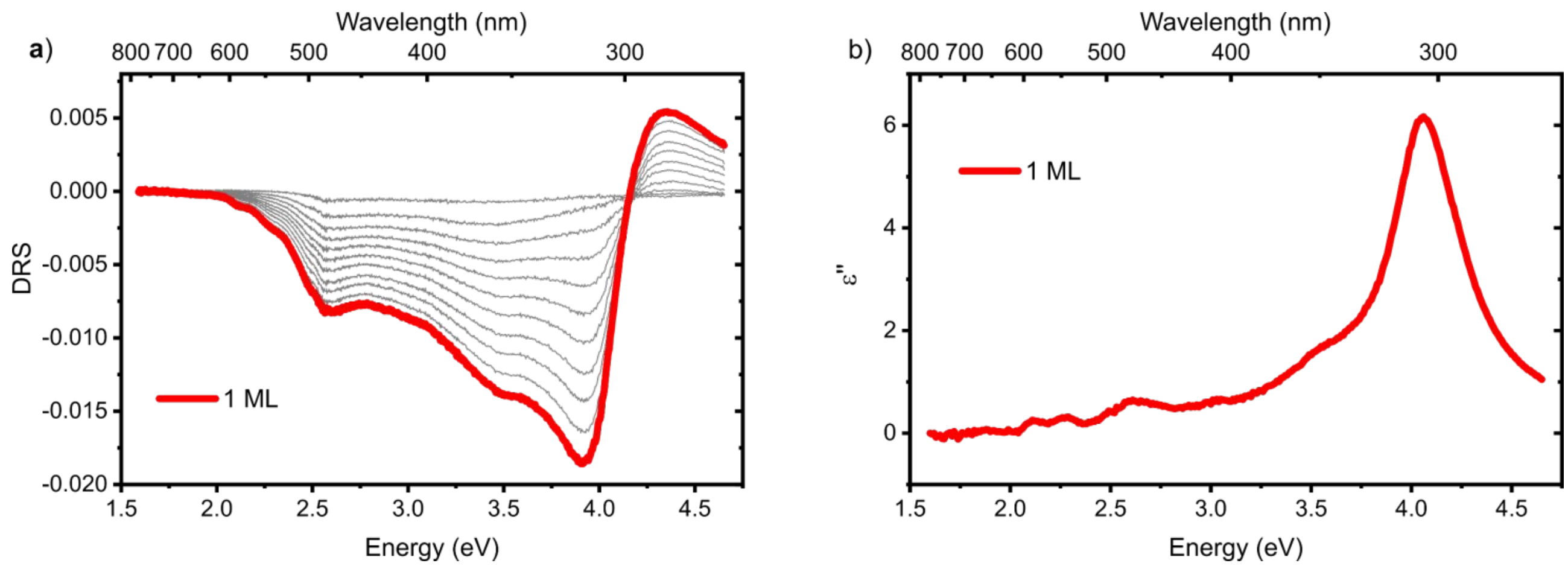

Figure S7 – a) In-situ DRS signal of pentacene on WS_2/Au(111) up to 1 ML (red line). The gray lines correspond to submonolayer coverages. b) Imaginary part of the numerically extracted complex dielectric function.

Fig. S5 a) shows the DRS signals acquired during the deposition of 5A on WS_2/Au(111). Up to 1 ML, the DRS signal increases while the spectral behavior does not significantly change. Right at the beginning, it is feasible that small areas of bare Au(111), e.g., holes in the WS_2 layer, are covered preferably, which could lead to the observed relatively smaller feature at 4 eV. For higher film thicknesses new transitions around 1.84 eV occur (not shown here), which we associate with the beginning of the 2nd layer growth and thus enabled us to determine the growth rate.

Since DRS signals do not only depend on the optical constants of the thin film but also largely on the optical constants of the substrate, an interpretation in terms of light absorption of the thin film can be difficult. We therefore extracted the thickness-dependent optical constants of the thin film numerically using the algorithm described in Ref. [3]. The imaginary part of the dielectric function ε'' is shown in Fig. S5 b). Noteworthily, we find that the spectral fingerprint clearly indicates the presence of 5A monomers within the 1st ML. These are characterized by the strong absorption band around 4 eV and a shoulder at around 3.5 eV, accompanied by small features at 2.12 eV and 2.28 eV, as similarly observed on a sapphire substrate[4]. This clearly indicates that the electronic and optical interaction of the molecules with the substrate, but also between molecules within the monolayer, can be considered to be very small. As argued in ref. [4], this optical behavior implies flat-lying molecules in the 1st ML. However, being DRS a photon-in/photon-out technique it is sensitive to only electric dipole allowed excitations, while it was demonstrated other interaction mechanisms between OSC-TMD interfaces, like the presence of dark excitons, normally undetectable with this technique[5,6].

It should be further mentioned that we interpret the small feature at 2.6 eV as an artifact because we neglect the WS_2 layer in our optical layer model. This was necessary because, to the best of our knowledge, precise optical constants of monolayer WS_2 interacting with the Au(111) surface are not available in the literature for the here considered spectral region. Except of this feature, the impact on the optical constants of

5A, however, is small, since a WS_2 ML changes the reflectance of the Au(111) surface only to a minor extent[7].

Bibliography

1. Black, E., Kratzer, P. & Morbec, J. M. Interaction between pentacene molecules and monolayer transition metal dichalcogenides. *Physical Chemistry Chemical Physics* **25**, 29444–29450 (2023).
2. Black, E. & Morbec, J. M. Effect of molecular rotation and concentration on the adsorption of pentacene molecules on two-dimensional monolayer transition metal dichalcogenides. *Electronic Structure* **6**, 025008 (2024).
3. Forker, R., Gruenewald, M. & Fritz, T. Optical differential reflectance spectroscopy on thin molecular films. *Annual Reports Section 'C' (Physical Chemistry)* **108**, 34 (2012).
4. Zhang, L., Fu, X., Hohage, M., Zeppenfeld, P. & Sun, L. D. Growth of pentacene on αAl2O3(0001) studied by in situ optical spectroscopy. *Phys. Rev. Mater.* **1**, 043401 (2017).
5. Bennecke, W. *et al.* Hybrid Frenkel–Wannier excitons facilitate ultrafast energy transfer at a 2D–organic interface. *Nat. Phys.* **21**, 1973–1980 (2025).
6. Katzer, M. *et al.* Impact of dark excitons on Förster-type resonant energy transfer between dye molecules and atomically thin semiconductors. *Phys. Rev. B* **107**, 035304 (2023).
7. Cho, E. *et al.* Thickness-dependent optical characteristics of WS 2 flakes prepared by Au- and Ag-assisted exfoliation. *J. Phys. D Appl. Phys.* **56**, 325101 (2023).
